# The microenvironment in canine mast cell tumors: association between tumor-associated tissue eosinophilia and histological grade

**DOI:** 10.3389/fvets.2026.1897478

**Published:** 2026-08-24

**Authors:** María Ezquerra-Durán, Pablo Cardenal-Morales, Luis Javier Ezquerra-Calvo, Raquel Tarazona-Lafarga, Massimo Santella, María Esther Durán-Flórez

**Affiliations:** 1 Small Animal Surgery Unit, Veterinary Teaching Hospital, Universidad de Extremadura, Caceres, Spain; 2 MINVET Research Group, Animal Medicine Department, Universidad de Extremadura, Caceres, Spain; 3 Surgery Unit, Animal Medicine Department, Universidad de Extremadura, Caceres, Spain; 4 Immunology Unit, Physiology Department, Universidad de Extremadura, Caceres, Spain; 5 Pathology Unit, Animal Medicine Department, Universidad de Extremadura, Caceres, Spain

**Keywords:** canine, eosinophils, hematological ratios, mast cell tumors, tumor-associated tissue eosinophilia

## Abstract

**Introduction:**

Canine mast cell tumors (MCTs) are common neoplasms with heterogeneous biological behavior, and histological grading alone may not fully reflect their aggressiveness. The tumor microenvironment, particularly tumor-associated tissue eosinophilia (TATE), may provide complementary information on MCT biology.

**Methods:**

This study evaluated whether intratumoral eosinophil infiltration, expressed as the eosinophil-to-mast cell ratio (EMR), was associated with histological grade in canine MCTs. A secondary objective was to assess the relationship between peripheral blood inflammatory markers and tumor grade. A total of 117 surgically excised MCTs from 73 dogs were included. Tumors were classified according to the Patnaik and Kiupel grading systems. Eosinophils and mast cells were counted in 10 consecutive high-power fields of neoplastic tissue, and the EMR was calculated. Histopathological variables, peripheral blood eosinophil counts, and inflammatory ratios were also evaluated. Univariable and multivariable regression models were used to assess associations with tumor grade, and receiver operating characteristic curve analysis was performed to identify an exploratory, data-derived EMR cutoff, which was internally validated using bootstrap resampling.

**Results:**

A higher EMR was associated with high-grade tumors in both grading systems and remained independently associated with tumor grade after adjustment for confounding variables. An exploratory EMR cutoff value of 0.38 showed good discriminatory ability for distinguishing low- from high-grade tumors according to Kiupel grading and showed stable overall discrimination on internal bootstrap validation, although the exact threshold remained imprecisely estimated. In contrast, peripheral blood eosinophil counts were not significantly related to tumor grade, and most systemic inflammatory markers showed no significant association with tumor grade.

**Discussion:**

These findings suggest that the local eosinophilic component of the tumor microenvironment, assessed through the EMR, may provide complementary information for the histological characterization of canine MCTs. Larger multicenter studies are needed to validate the proposed EMR cutoff and clarify its prognostic and biological relevance.

## 1 Introduction

Approximately 20% of cutaneous neoplasms diagnosed in dogs are mast cell tumors (MCTs) (1–3). Despite their high frequency, their etiology remains incompletely understood (4). MCTs are malignant round cell neoplasms with highly heterogeneous biological behavior, ranging from slow-growing solitary masses that can be controlled by surgery to rapidly growing, locally invasive or metastatic lesions that require a multimodal therapeutic approach and for which long-term control remains challenging (1, 5, 6).

Histologically, canine MCTs are classified using the Patnaik (7) and Kiupel (8) grading systems. The Patnaik system assigns tumors to three grades based on neoplastic cellularity, cellular morphology, mitotic index, extent of tissue involvement, and stromal reaction (7). In contrast, the Kiupel system proposes a binary classification, high or low grade, based on histological parameters such as mitotic index and the morphological features of neoplastic mast cells (8). However, there is no consensus as to which system is more useful across all clinical contexts (1), as the biological complexity of this neoplasm is not always fully captured by histological grading. This has encouraged the incorporation of additional diagnostic parameters, including immunohistochemical markers and assessment of the tumor microenvironment (TME) (9).

The TME comprises non-neoplastic elements that infiltrate and surround the tumor and, through continuous interactions with neoplastic cells, influence growth, invasiveness, metastatic potential, and therapeutic response (10, 11). This ecosystem includes the extracellular matrix, fibroblasts, blood vessels, immune cells, and multiple soluble mediators, such as cytokines, chemokines, and other signaling molecules (11). Moreover, the TME participates in key processes involved in tumor progression, including angiogenesis, immune evasion, and the activation of invasive and metastatic programs (1).

In veterinary oncology, characterization of the TME has attracted increasing interest, both because spontaneous tumors in dogs represent a valuable comparative model and because the immune landscape of many canine neoplasms remains less well characterized than in human medicine (1). A better understanding of the immune infiltrate and its regulatory mechanisms could improve prognostic stratification and support the development of more personalized therapeutic strategies. There has also been growing interest in the interaction between stromal components and immune mechanisms, as specific patterns of collagen organization and different forms of macrophage–extracellular matrix interaction have been associated with distinct outcomes in comparative oncology (8, 12).

Within the immune component of the TME, cellular infiltration may exert either antitumor or protumor functions depending on the local signaling context (2, 10, 13). In MCTs, infiltrates composed of immune cells of both myeloid and lymphoid origin have been described, and their characteristics may influence the balance between immune surveillance and tumor tolerance (8, 12). These infiltrates include effector populations, such as CD8 + T cells and CD4 + T cells with a predominantly Th1 profile, as well as immunosuppressive subpopulations, such as regulatory T cells (Tregs) (14). Likewise, tumor-associated macrophages (TAMs) can promote tumor progression, angiogenesis, and immunosuppression, and in canine tumors, subpopulations such as CD163+/CD204+ macrophages have been linked to aggressiveness, metastasis, and tumor-related death (15–17).

A morphological feature of the TME that has attracted particular interest is tumor-associated tissue eosinophilia (TATE) (18). Eosinophils may be recruited to neoplastic tissue by cytokines and chemokines produced by neoplastic and immune cells and, once activated, can release mediators capable of modulating tumor biology, including proangiogenic and remodeling effects (9). In canine MCTs, mast cells themselves promote the recruitment of eosinophils and other leukocyte populations, and mast cell–eosinophil interactions may establish feedback loops involving profibrotic and proangiogenic mediators (19). However, the role of eosinophils in cancer remains context-dependent, as their presence has been associated with better prognosis in some tumors (20, 21) and with more aggressive phenotypes in others (22, 23). Their potential contribution to angiogenesis suggests that, in certain contexts, TATE may reflect a microenvironment favorable to tumor progression and recurrence. In canine MCTs, high levels of TATE have been associated with poorer cellular differentiation, tumor neoangiogenesis, and increased risk of recurrence (9).

A potential connection between TATE and tumor-associated blood eosinophilia (TABE) has also been proposed (9). Variations in peripheral eosinophil counts may be accompanied by changes in hematological ratios involving this cell population, such as the neutrophil-to-eosinophil ratio (NER) or eosinophil-to-lymphocyte ratio (ELR), which provide an indirect approach to eosinophilic activation and systemic inflammation (24). Other routine hematological and biochemical markers have also gained interest in comparative oncology. Ratios such as the neutrophil-to-lymphocyte ratio (NLR), lymphocyte-to-monocyte ratio (LMR), platelet-to-lymphocyte ratio (PLR), and systemic immune-inflammation index (SII) have been proposed as indirect markers of the balance between antitumor immunity and protumor inflammation (25, 26). In MCTs, these ratios have been evaluated in relation to biological behavior and clinical outcome (24, 25), and a higher NLR has been associated with a higher histopathological grade (25). Likewise, the albumin-to-globulin ratio (AGR) may provide complementary information on systemic inflammation and the general condition of the patient (27, 28).

Finally, only a limited number of studies have characterized the immune TME of MCTs. Evidence focused specifically on TATE is even scarcer, as is evidence regarding its potential systemic expression through TABE and eosinophil-related hematological ratios (29). This knowledge gap hampers the understanding of the aggressive behavior of certain tumors and limits the identification of potential therapeutic targets (9).

The main objective of this study was to evaluate whether intratumoral eosinophil count and other histological parameters are associated with greater tumor aggressiveness in MCTs, as determined using the Patnaik and Kiupel grading systems. A secondary objective was to analyze the relationship between peripheral blood markers and the degree of mast cell tumor aggressiveness.

## 2 Materials and methods

### 2.1 Study population

A prospective analysis was conducted on tissue samples obtained from a retrospective cohort of dogs that underwent surgical excision of cutaneous and subcutaneous MCTs at the University Veterinary Hospital of Cáceres, Spain, between 2018 and 2024. Ethical approval was obtained from the Animal Experimentation Ethics Committee of the University of Extremadura, in accordance with Royal Decree 53/2013. The study was registered under approval number 77/2023, dated June 15, 2023.

### 2.2 Histopathological evaluation

Formalin-fixed paraffin-embedded (FFPE) tissue blocks were retrieved from the pathology archives. For cases processed during the study period, representative tissue samples were selected and embedded in paraffin wax according to routine histopathological procedures. Sections of 5 μm thickness were prepared and stained with hematoxylin and eosin (H&E), toluidine blue, and Masson’s trichrome. All slides were evaluated by the same senior pathologist.

Histopathological parameters were assessed to classify MCTs according to their degree of malignancy using the grading systems described by Patnaik (7) and Kiupel (8). Surgical margins smaller than 5 mm were considered incomplete (30, 31). Histopathological parameters were assessed to classify MCTs according to their degree of malignancy using the grading systems described by Patnaik (7) and Kiupel (8).

TATE was assessed by counting manually eosinophils and mast cells in 10 consecutive high-power fields (HPFs) of neoplastic tissue, avoiding areas with secondary changes that could interfere with the evaluation. The total area analyzed per biopsy was 2.38 mm^2^. Cell counts were performed on H&E-stained sections after confirming the presence of mast cell tumors (MCTs) by toluidine blue staining. Degranulated mast cells were also included in the cell counts. The mean count for each cell population was calculated, and the eosinophil-to-mast cell ratio (EMR) was determined using the following formula: EMR = mean eosinophil count/mean mast cell count.

Histological parameters, including necrosis, fibrosis, and tissue inflammation, as well as the intensity of cytoplasmic granulation in mast cells and eosinophils, were assessed subjectively based on the evaluator’s experience. Necrosis, fibrosis, and tissue inflammation were classified as low when they involved <10% of the analyzed area, moderate when involvement ranged from 10 to 25%, and extensive when >25% of the analyzed area was affected. Intracytoplasmic granulation of mast cells and eosinophils was classified into three categories. Granulation was considered high when most cells, defined as ≥75%, showed a high density of secretory granules that obscured cytoplasmic spaces and, in many cases, the nucleus. It was classified as moderate when most cells, defined as ≥75%, showed a marked concentration of cytoplasmic granules while still displaying multiple cytoplasmic areas devoid of granules. Granulation was considered low when most cells, defined as ≥75%, showed only occasional secretory granules, which were sometimes difficult to detect. For mast cells, an additional category, absent, was assigned when intracytoplasmic granulation could not be detected in any of the observed cells.

### 2.3 Peripheral blood inflammatory biomarkers and derived indices

Preoperative hematological results obtained as part of the routine clinical assessment were retrospectively retrieved from the medical records. The variables included total leukocyte count, differential leukocyte counts, including neutrophils, eosinophils, lymphocytes, and monocytes, and platelet count. All hematological analyses had been performed using an automated hematology analyzer (Element HT5™; Antech Diagnostics, USA).

Biochemical variables included albumin, total protein, and serum calcium concentrations. Globulin concentration was estimated as the difference between total protein and albumin concentrations. Biochemical analyses were performed using an automated analyzer (Spin200E; Spinreact®, Spain).

Inflammatory biomarker ratios were calculated using absolute cell counts and serum protein concentrations as follows: neutrophil-to-lymphocyte ratio (NLR), eosinophil-to-lymphocyte ratio (ELR), neutrophil-to-eosinophil ratio (NER), lymphocyte-to-monocyte ratio (LMR), platelet-to-lymphocyte ratio (PLR), systemic immune-inflammation index (SII), calculated as platelet count × neutrophil count/lymphocyte count, and albumin-to-globulin ratio (AGR).

Absolute hematological counts were expressed as 10^3^/μL, whereas albumin, globulins, and total protein concentrations were expressed as g/dL. All calculated ratios were considered dimensionless.

In dogs with multiple synchronous tumors of different histological grades, the peripheral blood sample was assigned only to the tumor with the highest histological grade to evaluate whether inflammatory biomarkers were associated with more aggressive disease.

### 2.4 Statistical analysis

Descriptive statistics were used to summarize patient and tumor characteristics. Continuous variables were expressed as mean and standard deviation (SD), and categorical variables as frequencies and percentages.

The associations between histopathological variables, inflammatory biomarkers, and tumor grade according to the Kiupel grading system were first explored using univariable logistic regression analyses.

A multivariable logistic regression model was constructed to assess the association between the EMR and tumor grade according to the Kiupel grading system while accounting for potential confounding. Potential confounders were selected according to epidemiological criteria; specifically, variables were considered potential confounders if they were associated with the exposure, were independent risk factors for the outcome, and were not part of the causal pathway. During model construction, confounding variables were retained if their inclusion produced a change of at least 10% in the effect estimate of the main exposure variable.

Model selection was based on statistical criteria, clinical coherence, and parsimony. Collinearity between covariates was assessed using variance inflation factors (VIFs). Results were reported as odds ratios (ORs) with 95% confidence intervals (95% CIs). Because some ORs were extreme and generated asymmetric CIs, regression coefficients were additionally presented on the logarithmic scale as log-odds.

Given the large effect estimates and wide confidence intervals observed in the conventional maximum-likelihood logistic regression model, the final Kiupel model was additionally evaluated using Firth’s penalized logistic regression as a sensitivity analysis to reduce potential small-sample and sparse-data bias and to provide more stable estimates in the presence of possible quasi-separation.

Receiver operating characteristic (ROC) curve analysis was performed to evaluate the discriminative ability of the EMR for distinguishing Kiupel low- and high-grade tumors. An exploratory, data-derived cutoff was identified by maximizing the Youden index, and the area under the curve (AUC), sensitivity, and specificity were calculated. Internal validation of the exploratory EMR cutoff was performed using nonparametric bootstrap resampling with 2,000 replicates. In each bootstrap sample, 117 tumors were sampled with replacement from the original dataset, and the EMR cutoff maximizing the Youden index was recalculated. The bootstrap distributions of the optimal cutoff, AUC, sensitivity, and specificity were examined. Because the distribution of the bootstrap-derived cutoff was asymmetric, percentile-based bootstrap intervals were used to describe its variability.

To assess the association between the EMR and tumor grade according to the Patnaik grading system, multivariable ordinal logistic regression models were constructed using the same methodological approach as that applied to the Kiupel models, including univariable screening, assessment of confounding and collinearity, and model selection based on statistical criteria, clinical coherence, and parsimony. The proportional odds (parallel-lines) assumption was formally assessed globally and for individual predictors using a generalized ordered logit framework. When all predictors satisfied the proportional odds assumption, a standard proportional odds ordinal logistic regression model was retained. When the assumption was violated by one or more predictors, a partial proportional odds model was fitted, allowing the coefficients of variables violating the assumption to vary across cumulative logits while maintaining the proportional odds constraint for variables satisfying the assumption.

All statistical analyses were performed using StataBE v.17 (StataCorp, College Station, TX, USA). A two-sided *p*-value < 0.05 was considered statistically significant.

## 3 Results

A total of 73 dogs and 117 MCTs were included in the study. Overall, 31.5% of the dogs developed multiple MCTs, either synchronously or metachronously. The majority of MCTs were cutaneous (89.7%). The mean age at presentation was 8.9 years, 49.3% of the patients were male, and 23.3% were mixed-breed dogs.

According to the histopathological grading system described by Kiupel (8), 53% of MCTs were classified as high grade. In contrast, when classified according to the system described by Patnaik (7), 48.7% of MCTs were assigned to the intermediate grade (grade II). Of the 57 MCTs classified as Patnaik grade II, 34 (59.7%) were classified as high grade according to the Kiupel system (Table 1).

**Table 1:** General description of mast cell tumors.

*N* (%)	117 (100)
Mast cell tumor type, *N* (%)	
Cutaneous	105 (89.7)
Subcutaneous	12 (10.3)
Size ≥ 3 cm. *N* (%)	23 (19.7)
Kiupel system, *N* (%)	
Low grade	55. (47.0)
High grade	62 (53.0)
Patnaik	
Grade I	32 (27.4)
Grade II	57 (48.7)
Grade III	28 (23.9)

### 3.1 Histopathological evaluation

#### 3.1.1 Kiupel grading system

**Figure 1:**
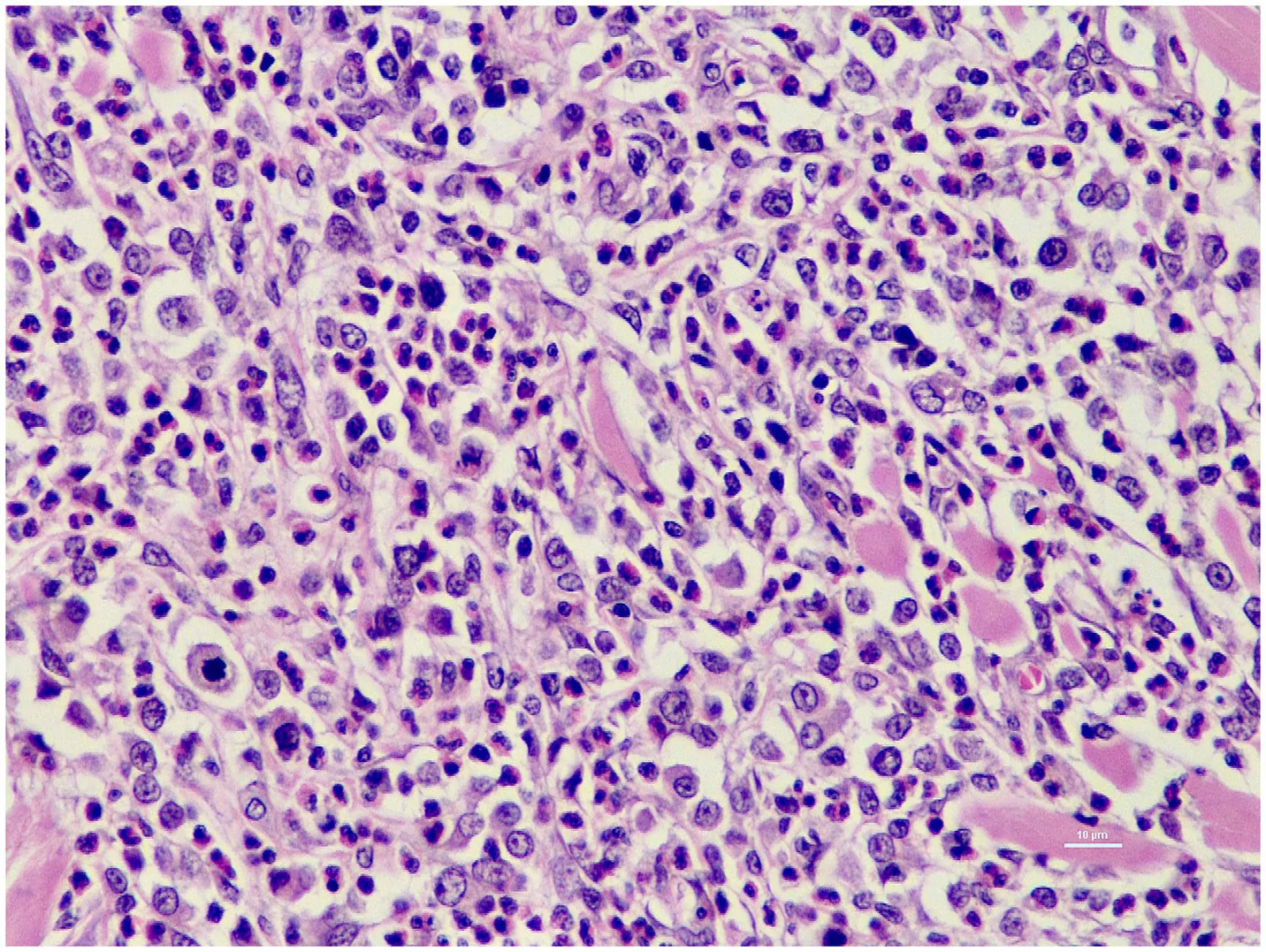
High-grade mast cell tumor according to the Kiupel system, showing marked tissue eosinophilia. Hematoxylin and eosin, ×400.

**Figure 2:**
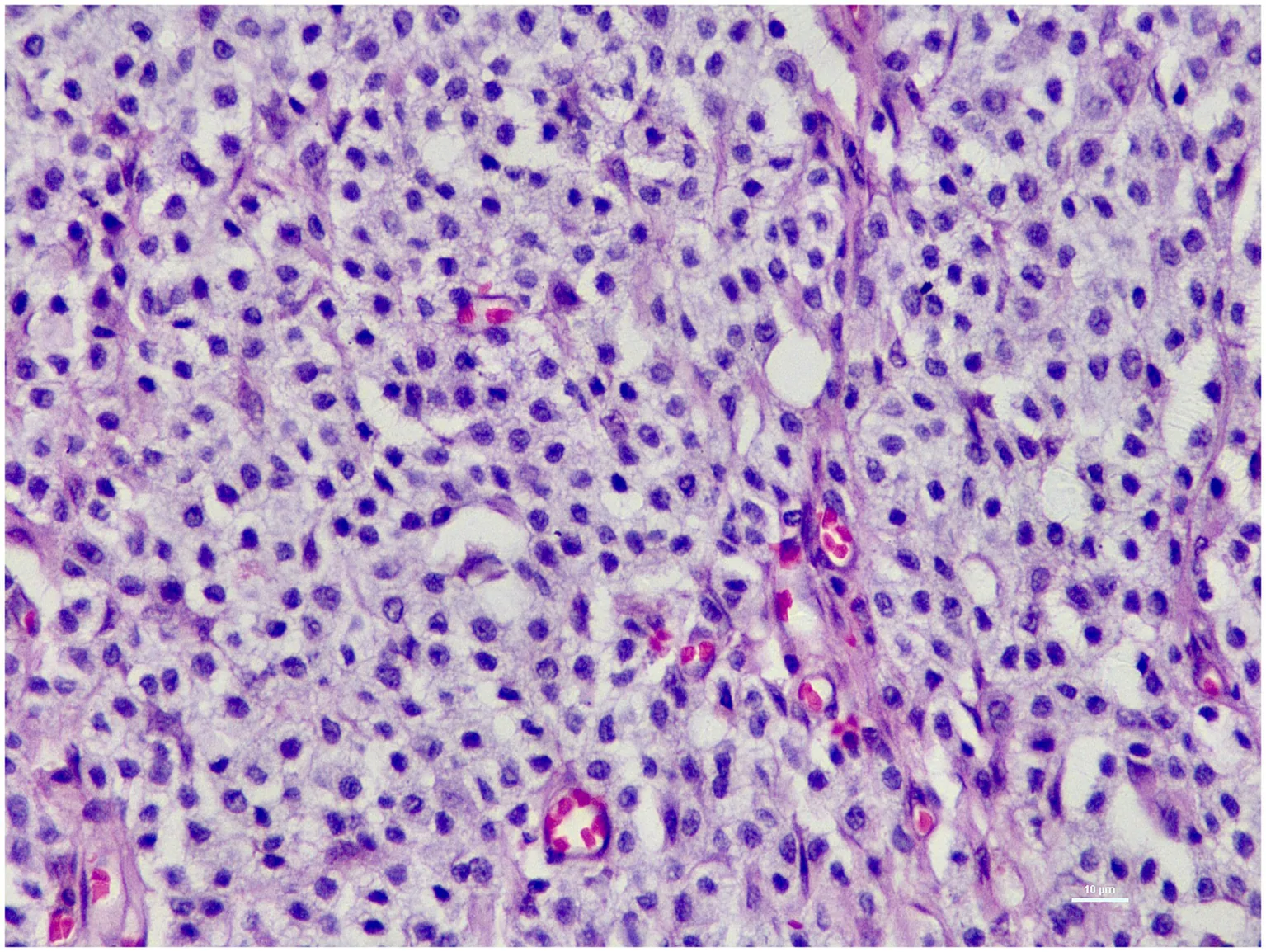
Low-grade mast cell tumor according to the Kiupel system, showing near absence of eosinophils in the tumor microenvironment. Hematoxylin and eosin, ×400.

**Figure 3:**
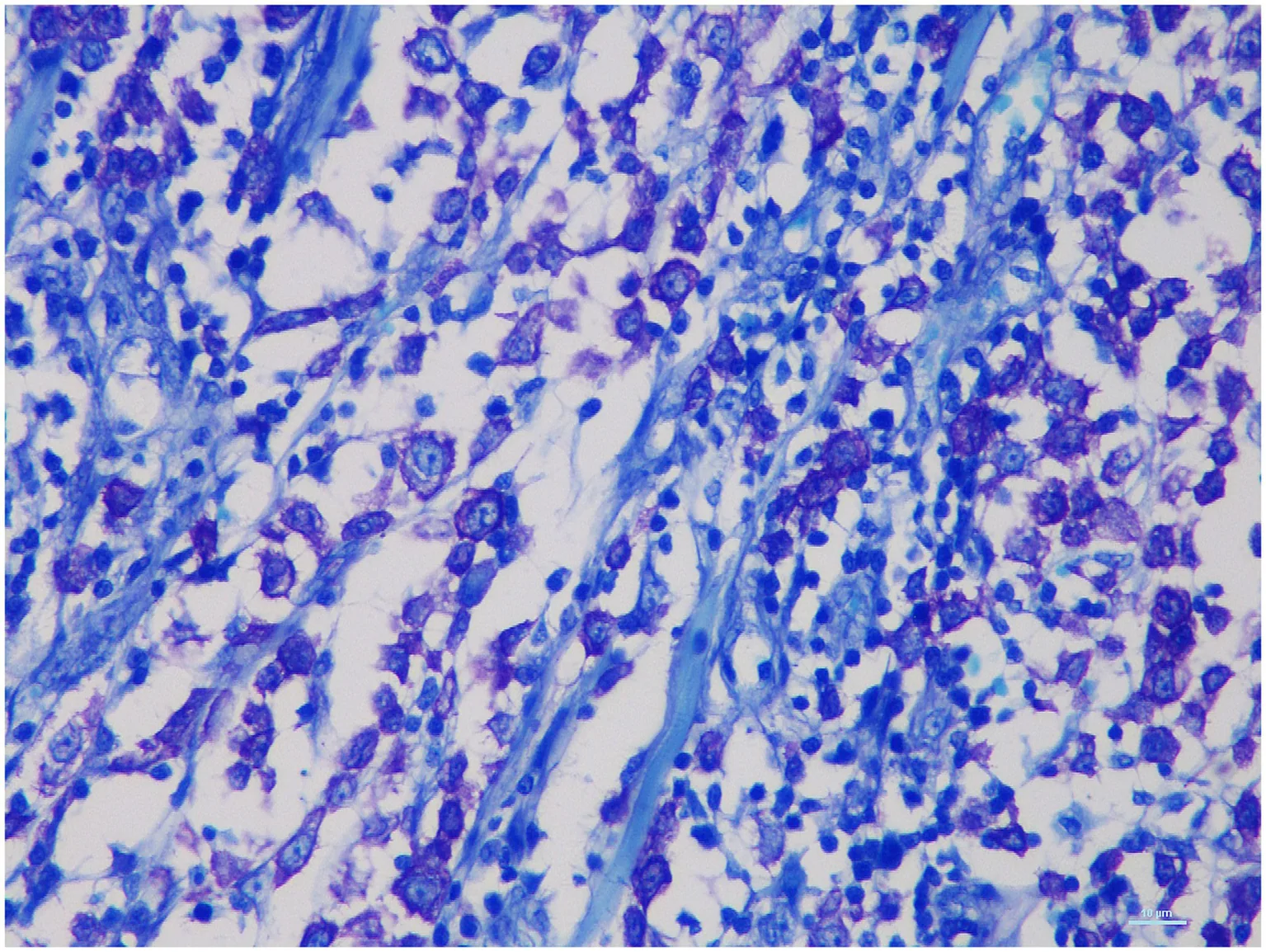
Metachromasia of the secretory granules in neoplastic mast cells from a high-grade mast cell tumor (Kiupel grading system). The cytoplasmic granules are present in low to moderate numbers. Toluidine blue stain, ×400.

In the univariable analysis, the presence of moderate to extensive necrosis (OR 4.2 [95% CI 1.1–15.6]), mitotic index (OR 1.7 [95% CI 1.1–2.4]), vascular invasion (OR 6.3 [95% CI 2.2–18.1]), and EMR (OR 35.3 [95% CI 8.3–149.3]) were positively associated with high-grade tumors according to the Kiupel grading system. In contrast, moderate and high mast cell granulation were negatively associated with high-grade tumors (OR 0.1 [95% CI 0.0–0.5] and OR 0.1 [95% CI 0.0–0.2], respectively). The remaining associations between histopathological parameters and Kiupel grade are shown in Table 2. Representative histological images of high-grade and low-grade MCTs with different degrees of tissue eosinophilia are shown in Figures 1, 2. Figure 3 shows a representative photomicrograph of a high-grade mast cell tumor (Kiupel grading system) stained with toluidine blue.

**Table 2:** Descriptive analysis of the histological variables evaluated according to histological grade using the Kiupel classification.

*N* = 117 (100%)	Low grade 55 (47%)	High grade 62 (53%)	OR (IC 95%)	*p* value
Cutaneous MCT, *N* (%)	47 (85.5)	58 (93.6)	2.47 (0.70–8.70)	0.160
MCT ≥ 3 cm, *n* (%)	10 (18.2)	13 (21.0)	1.20 (0.48–3.0)	0.705
Tumor cellularity, *n* (%)				
Low (ref)	2 (3.6)	5 (8.1)	–	
Moderate	13 (23.6)	23 (37.1)	0.71 (0.12–4.18)	0.703
High	40 (72.7)	34 (54.8)	0.34 (0.06–1.87)	0.214
Moderate/extensive necrosis, *n* (%)	3 (5.5)	12 (19.4)	4.16 (1.11–15.63)	**0.035**
Mitotic index, mean (SD)	0.3 (1.0)	1.4 (2.5)	1.65 (1.11–2.43)	**0.013**
Mast cell granulation, *n* (%)				
Absent or low (ref)	3 (5.5)	26 (41.9)	–	
Moderate	21 (38.2)	22 (35.5)	0.12 (0.03–0.46)	**0.002**
High	31 (56.4)	14 (22.6)	0.05 (0.01–0.20)	**<0.001**
Eosinophil granulation, *n* (%)				
Low (ref)	10 (18.2)	20 (32.8)	–	
Moderate	24 (43.6)	21 (34.4)	0.44 (0.17–1.14)	0.091
High	21 (38.2)	20 (32.8)	0.48 (0.18–1.26)	0.136
Moderate/extensive inflammation^*^, *n* (%)	6 (10.9)	14 (22.6)	2.38 (0.85–6.71)	0.101
Fibrosis, *n* (%)				
Low (ref)	11 (20.0)	13 (21.0)	–	
Moderate	14 (25.5)	14 (22.6)	0.85 (0.28–2.52)	0.764
Extensive	30 (50.6)	35 (56.5)	0.99 (0.39–2.53)	0.979
EMR, mean (SD)	0.3 (0.3)	0.7 (0.5)	35.29 (8.34–149.31)	<0.001
EMR ≥ 0.38, *n* (%)^**^	13 (23.6)	49 (79.0)	12.18 (5.09–29.13)	**<0.001**
Vascular invasion, *n* (%)	5 (9.1)	24 (38.7)	6.32 (2.21–18.08)	**0.001**
Lymph node involvement, *n* (%)	2 (4.8)	4 (7.6)	1.63 (0.28–9.38)	0.583
Incomplete margin, *n* (%)	32 (58.2)	46 (74.2)	2.07 (0.96–4.51)	0.069

^*^Dichotomous inflammation: absent/low vs moderate/extensive.

^**^Exploratory, data-derived cutoff selected by maximizing the Youden index in the original cohort; this threshold requires external validation.

MCT, Mast cell tumor; SD, Standard Deviation; Ref, Reference; EMR, Eosinophil-to-Mast cell-Ratio. Bold values represent statistically significant differences.

**Figure 4:**
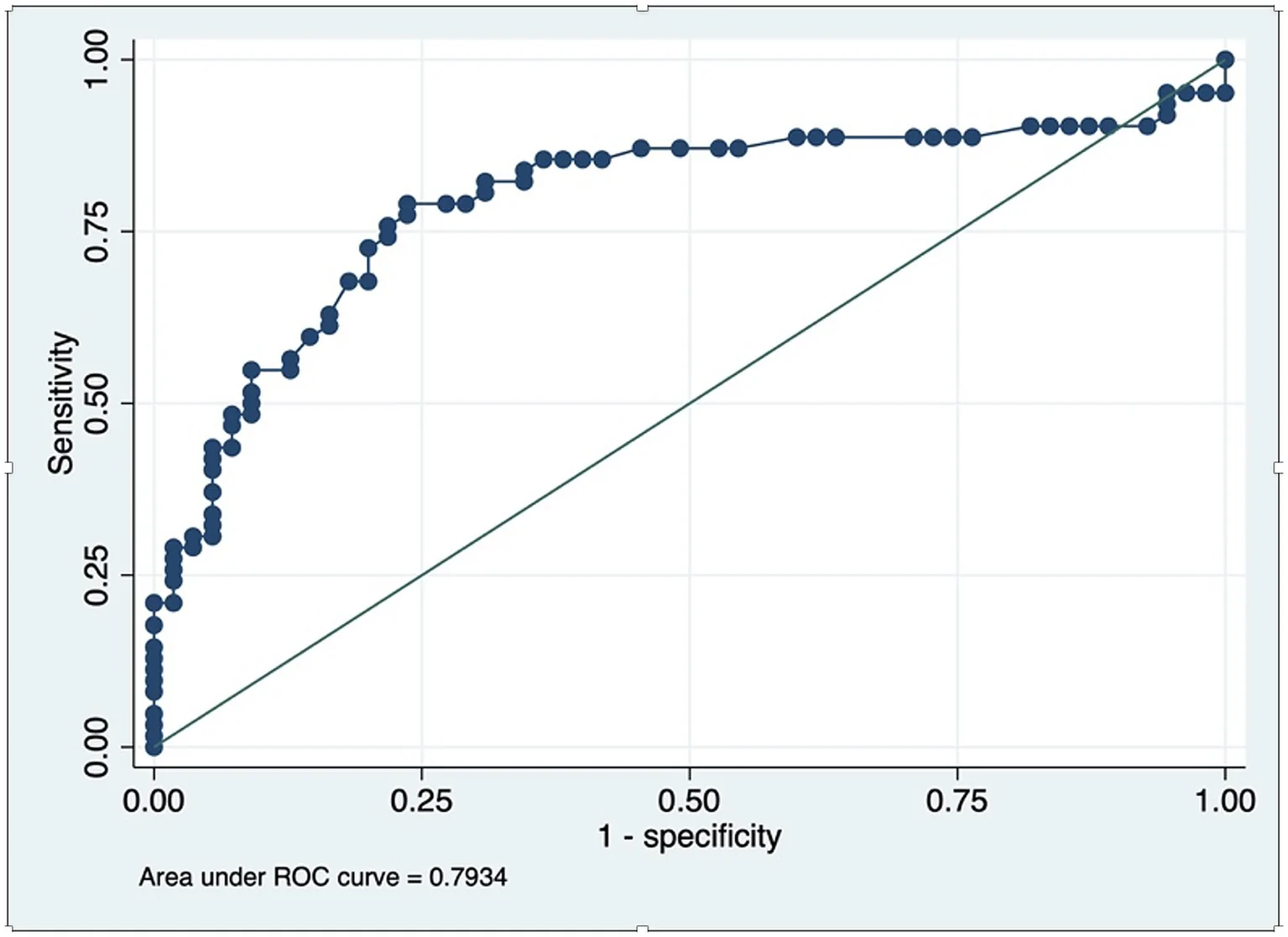
Receiver operating characteristic (ROC) curve showing the diagnostic performance of eosinophil-to-mast cell ratio for predicting high grade in Kiupel system.

An exploratory EMR cutoff of 0.38 showed the highest Youden index for distinguishing low-grade from high-grade tumors according to the Kiupel system, with an apparent AUC of 0.79 (95% CI 0.71–0.86), sensitivity of 79.0%, specificity of 76.4%, positive predictive value of 79.0%, and negative predictive value of 76.4% (Figure 4). Internal validation using 2,000 nonparametric bootstrap replicates yielded a median optimal cutoff of 0.38 (95% percentile interval, 0.29–0.63). The median bootstrap sensitivity and specificity were 78.9% (95% percentile interval, 58.3–91.0%) and 78.8% (95% percentile interval, 64.6–93.5%), respectively. The median bootstrap AUC was 0.796 (95% percentile interval, 0.705–0.878), indicating relatively stable discriminatory performance under resampling. However, the variability observed in the bootstrap distribution of the optimal threshold indicates that 0.38 should be regarded as an exploratory, data-derived cutoff requiring external validation.

**Figure 5:**
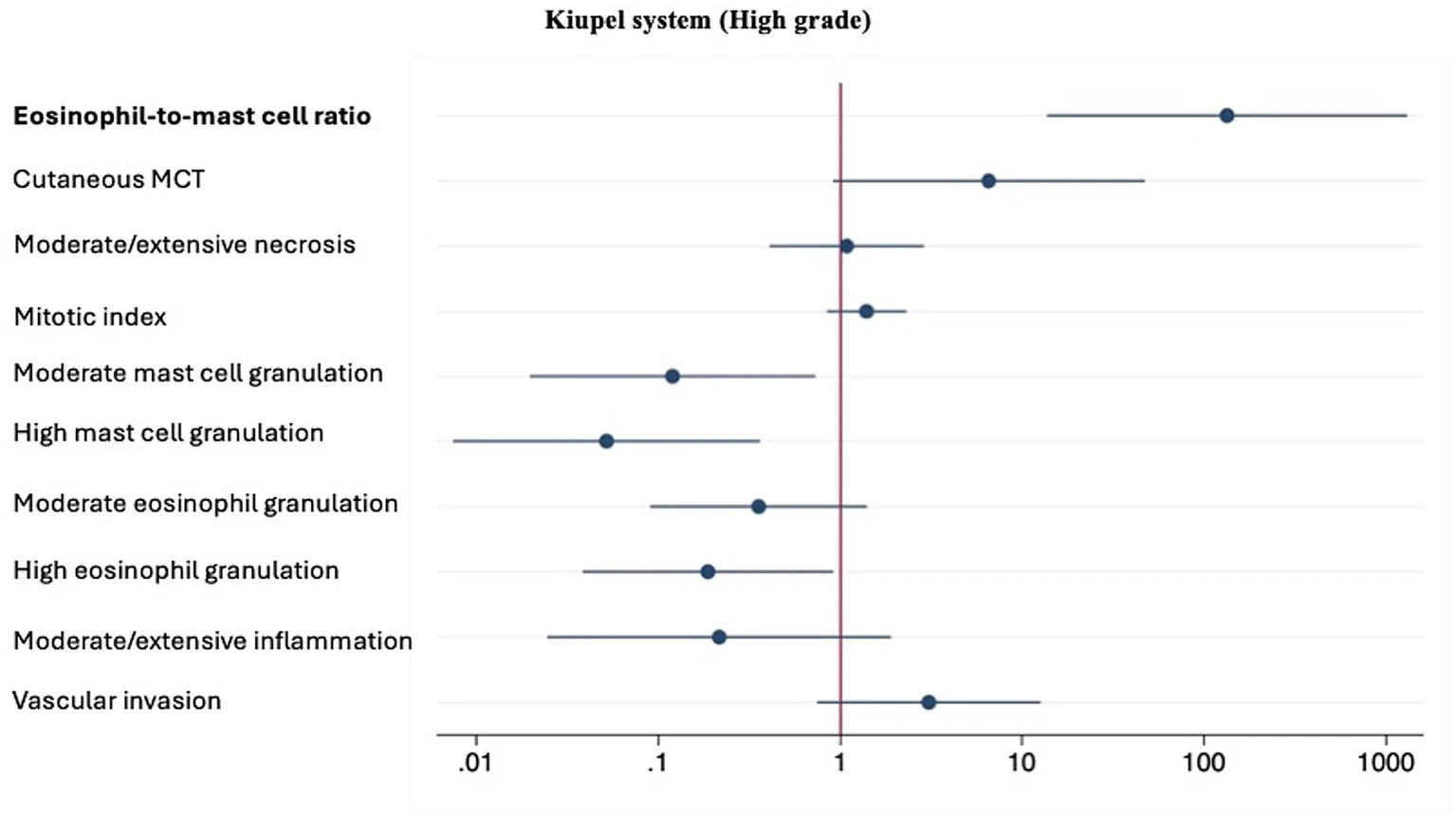
Coefficient plot from the adjusted logistic regression model for high-grade classification according to the Kiupel system.

After adjustment for confounding variables, EMR remained independently associated with high-grade tumors (OR 133.7 [95% CI 13.7–1301.5]). The final multivariable model, including EMR and the selected confounding variables, is shown in Table 3 and Figure 5. No relevant collinearity was detected, as all variables included in the model had VIF values <3.

**Table 3:** Final model. Logistic regression model evaluating the effect of the eosinophil-to-mast cell ratio in the presence of confounding and interaction factors using the Kiupel system.

Kiupel system (high grade)	OR (IC 95%)	*p* value
Eosinophil-to-mast cell ratio	133.71 (13.74–1301.46)	<0.001
Cutaneous MCT	6.56 (0.91–47.35)	0.062
Moderate/extensive necrosis	1.08 (0.41–2.89)	0.871
Mitotic index	1.40 (0.84–2.32)	0.197
Mast cell granulation (ref. absent/low)		
Moderate	0.12 (0.02–0.73)	0.021
High	0.05 (0.01–0.36)	0.003
Eosinophil granulation (ref. absent)		
Moderate	0.36 (0.09–1.40)	0.140
High	0.19 (0.04–0.91)	0.038
Moderate/extensive inflammation	0.22 (0.02–1.90)	0.167
Vascular invasion	3.07 (0.74–12.64)	0.121

MCT, Mast cell tumor; Ref, Reference.

Because the conventional model yielded a very large effect estimate with a wide confidence interval, the analysis was repeated using Firth’s penalized logistic regression. EMR remained strongly and independently associated with Kiupel high-grade classification (OR 64.99 [95% CI 8.52–495.88]; *p* < 0.001). Although the penalized estimate was lower than the conventional maximum-likelihood estimate, the direction and statistical significance of the association were preserved, supporting the robustness of the association while indicating that the original effect estimate was likely somewhat inflated and should be interpreted cautiously.

#### 3.1.2 Patnaik grading system

As observed with the Kiupel grading system, moderate to extensive necrosis (OR 22.5 [95% CI 5.7–87.9]), mitotic index (OR 2.2 [95% CI 1.5–3.3]), vascular invasion (OR 9.9 [95% CI 4.0–24.6]), and EMR (OR 5.0 [95% CI 2.2–11.7]) were positively associated with higher-grade tumors according to the Patnaik system, whereas moderate and high mast cell granulation were negatively associated with higher-grade tumors (OR 0.1 [95% CI 0.0–0.3] and OR 0.1 [95% CI 0.0–0.2], respectively). However, unlike in the Kiupel system, larger MCTs (size ≥3 cm) (OR 2.9 [95% CI 1.2–7.3]), lymph node involvement (OR 7.6 [95% CI 1.3–42.5]), and incomplete surgical margins (OR 3.2 [95% CI 1.5–6.9]) were also associated with higher-grade tumors (Table 4).

**Table 4:** Descriptive analysis of the histological variables evaluated according to histological grade using the Patnaik classification.

*N* = 117 (100%)	Grade I 32 (27.4%)	Grade II 57 (48.7%)	Grade III 28 (23.9%)	OR (IC 95%)	*p* value
Cutaneous MCT, *n* (%)	25/78.1	56 (98.3)	24 (85.7)	2.37 (0.65–8.61)	0.191
MCT ≥ 3 cm, *n* (%)	5 (15.6)	7 (12.3)	11 (39.3)	2.90 (1.16–7.26)	**0.023**
Tumor cellularity, *n* (%)					
Low (ref)	1 (3.1)	6 (10.5)	0 (0)	–	
Moderate	10 (31.3)	19 (33.3)	7 (25.0)	2.09 (0.27–4.35)	0.902
High	21 (65.6)	32 (56.1)	21 (75.0)	1.35 (0.36–5.05)	0.656
Moderate/extensive necrosis, *n* (%)	0 (0)	3 (5.3)	12 (42.9)	22.47 (5.74–87.90)	**<0.001**
Mitotic index, mean (SD)	0.1 (0.3)	0.4 (1.0)	2.7 (3.1)	2.21 (1.49–3.27)	**<0.001**
Mast cell granulation, *n* (%)					
Absent or low (ref)	2 (6.3)	9 (15.8)	18 (64.3)	–	
Moderate	13 (40.6)	22 (38.6)	8 (28.6)	0.12 (0.04–0.34)	**<0.001**
High	17 (53.1)	26 (45.6)	2 (7.1)	0.06 (0.02–0.18)	**<0.001**
Eosinophil granulation, *n* (%)					
Low (ref)	6 (18.8)	17 (29.8)	8 (28.6)	–	
Moderate	15 (46.9)	21 (36.8)	9 (32.1)	0.59 (0.25–1.40)	0.234
High	11 (34.4)	19 (33.3)	11 (39.3)	0.85 (0.35–2.03)	0.713
Moderate/extensive Inflammation, *n* (%)^*^	3 (9.4)	5 (8.8)	12 (42.9)	5.89 (2.13–16.28)	**0.001**
Fibrosis, *n* (%)					
Low (ref)	7 (21.9)	10 (17.5)	7 (25.0)	–	
Moderate	7 (21.9)	12 (21.1)	9 (32.1)	1.22 (0.43–3.48)	0.714
Extensive	18 (56.3)	35 (61.4)	12 (42.9)	0.79 (0.32–1.93)	0.606
Eosinophil-to-mast cell ratio, mean (SD)	0.3 (0.2)	0.5 (0.5)	0.7 (0.5)	5.0 (2.2–11.7)	<0.001
Vascular invasion, *n* (%)	0 (0)	13 (22.8)	16 (57.1)	9.88 (3.97–24.60)	**<0.001**
Lymph node involvement, *n* (%)	0 (0)	2 (4.4)	4 (16.7)	7.55 (1.34–42.49)	**0.022**
Incomplete margin, *n* (%)	14 (43.8)	42 (73.7)	22 (78.6)	3.19 (1.48–6.87)	**0.003**

^*^Dichotomous inflammation: absent/low vs moderate/extensive.

MCT, Mast cell tumor; SD, Standard Deviation; Ref, Reference. Bold values represent statistically significant differences.

Two final models were retained because both were methodologically coherent, clinically interpretable, and consistent with the predefined confounding strategy. In model 1, the proportional odds assumption was satisfied (global Wald test of parallel lines: *χ*^2^ = 7.05, df = 5, *p* = 0.217), and no individual predictor showed evidence of violating this assumption, including EMR (*p* = 0.425). The standard proportional odds ordinal logistic regression model was therefore retained, and EMR remained independently associated with higher Patnaik grade (OR 5.58 [95% CI 2.10–14.78]; *p* = 0.001) (Table 5). In model 2, the proportional odds assumption was satisfied for EMR, necrosis, and vascular invasion, but not for tumor type (*p* < 0.001). Consequently, model 2 was re-estimated using a partial proportional odds model, allowing the effect of tumor type to vary across cumulative logits while retaining proportional odds constraints for the remaining predictors. In this revised model, EMR remained independently associated with higher Patnaik grade (common OR 7.55 [95% CI 2.61–21.81]; *p* < 0.001) (Table 6).

**Table 5:** Final model 1.

Patnaik system	OR (IC 95%)	*p* value
Eosinophil-to-mast cell ratio	5.58 (2.10–14.78)	0.001
MCT ≥ 3 cm	3.10 (1.08–8.78)	0.035
Mast cell granulation (ref. absent/low)		
Moderate	0.15 (0.05–0.46)	0.001
High	0.10 (0.03–0.31)	<0.001
Vascular invasion	7.98 (2.86–22.27)	<0.001

MCT, Mast cell tumor; Ref, Reference.

Proportional odds ordinal logistic regression model evaluating the association between the eosinophil-to-mast cell ratio and Patnaik histological grade.

**Table 6:** Final model 2.

Patnaik system	OR (IC 95%)	*p* value
Eosinophil-to-mast cell ratio	7.55 (2.61–21.81)	<0.001
Cutaneous MCT		
Grade II/III vs. Grade I	3.54 (0.88–14.19)	0.075
Grade III vs. Grade I/II	0.06 (0.01–0.37)	0.002
Moderate/extensive necrosis	80.00 (12.86–497.84)	<0.001
Vascular invasion	24.57 (5.86–103.04)	<0.001

MCT, Mast cell tumor.

Partial proportional odds model evaluating the association between the eosinophil-to-mast cell ratio and Patnaik histological grade.

### 3.2 Inflammatory biomarkers in peripheral blood

In an exploratory analysis of peripheral blood biomarkers and inflammatory ratios, most evaluated parameters showed no significant relationship with MCT grade according to either grading system (Table 7). Using the Kiupel grading system, no statistically significant differences were observed between low- and high-grade MCTs. Although NLR was numerically higher in high-grade tumors, this difference did not reach statistical significance.

**Table 7:** Peripheral blood markers in different grades of MCT.

Kiupel system				
*N* = 117 (100%)	Low grade 55 (47%)	High grade 62 (53%)	OR (IC 95%)	*p* value
Peripheral blood eosinophils (×10^3^/μl)	0.366 (0.384)	0.353 (0.326)	1.0 (1.0–1.0)	0.874
NLR	4.4 (2.6)	7.1 (7.2)	1.1 (1.0–1.3)	0.088
ELR	0.2 (0.2)	0.2 (0.2)	1.3 (0.1–19.8)	0.827
NER	105.0 (261.8)	41.9 (45.7)	1.0 (1.0–1.0)	0.222
LMR	5.1 (6.5)	4.9 (4.1)	1.0 (0.9–1.1)	0.856
PLR	182.4 (89.0)	229.4 (205.5)	1.0 (1.0–1.0)	0.255
SII	1281.1 (1327.8)	1674.6 (1674.9)	1.0 (1.0–1.0)	0.293
Albumin-to-globulin ratio	1.0 (0.4)	1.1 (0.3)	2.0 (0.4–9.0)	0.384
Calcium (mg/dL)	10.9 (1.0)	10.8 (1.1)	1.0 (0.5–1.7)	0.920

Grade Patnaik					
*N* = 117 (100%)	Grade I 32 (27.4%)	Grade II 57 (48.7%)	Grade III 28 (23.9%)	OR (IC 95%)	*p* value
Peripheral blood eosinophils (×10^3^/μl)	0.403 (0. 425)	0.350 (0. 309)	0.333 (0. 350)	1.0 (1.0–1.0)	0.521
NLR	4.0 (2.0)	6.1 (5.9)	7.6 (7.6)	1.1 (1.0–1.2)	0.071
ELR	0.2 (0.2)	0.2 (0.2)	0.2 (0.1)	0.8 (0.1–8.4)	0.868
NER	69.1 (177.7)	82.6 (214.8)	41.0 (30.7)	1.0 (1.0–1.0)	0.605
LMR	4.2 (3.5)	5.1 (5.9)	5.4 (5.2)	1.0 (0.9–1.1)	0.490
PLR	157.6 (56.9)	207.2 (155.8)	259.5 (233.4)	1.0 (1.0–1.0)	0.070
SII	943.6 (565.0)	1441.9 (1598.5)	2106.2 (1842.6)	1.0 (1.0–1.0)	**0.028**
Albumin-to-globulin ratio	1.0 (0.3)	1.1 (0.4)	1.1 (0.3)	1.3 (0.4–4.6)	0.695
Calcium (mg/dL)	10.5 (0.8)	11.0 (0.9)	11.0 (1.5)	1.5 (0.8–2.6)	0.202

MCT, Mast cell tumor; NLR, neutrophil-to-lymphocyte ratio; ELR, eosinophil-to-lymphocyte ratio; NER, neutrophil-to-eosinophil ratio; LMR, lymphocyte-to-monocyte ratio; PLR, platelet-to-lymphocyte ratio; SII, systemic immune-inflammation index. Values are presented as mean and Standard Deviation (SD). Bold values represent statistically significant differences.

When tumors were evaluated according to the Patnaik grading system, some inflammatory indices showed a progressive increase with increasing histological grade. NLR increased from grade I to grade III, although this trend was not statistically significant. Similarly, PLR was higher in grade III tumors, with a *p*-value close to statistical significance. SII showed a significant association with Patnaik grade, with progressively higher values from grade I to grade III, although the corresponding OR was close to 1. Peripheral blood eosinophil counts and eosinophil-related ratios did not show a significant association with either grading system.

## 4 Discussion

Canine mast cell tumor is one of the most common cutaneous neoplasms in dogs. Based on available data, cutaneous mast cell tumors account for approximately 20% of canine skin tumors and around 7% of all canine neoplasms (3, 32–34).

In the present study, the mean age at diagnosis was 8.9 years, consistent with previous reports indicating that this neoplasm occurs more frequently in middle-aged to older dogs (35–37). The influence of sex and reproductive status on the development and malignancy of canine mast cell tumors remains unclear. Although some studies have reported a higher risk in females (36), others have not identified an evident sex predisposition (35, 37). In our cohort, neither sex nor reproductive status was associated with histological aggressiveness.

According to tumor location, 89.7% of the MCTs analyzed were cutaneous, whereas 10.3% were subcutaneous. This distribution is consistent with previous reports, in which cutaneous MCTs clearly predominate over subcutaneous MCTs (38). MCTs were histologically classified using the Patnaik (7) and Kiupel (8) grading systems. Among the 57 tumors classified as Patnaik grade II, 59.7% (34/57) were classified as high grade according to the Kiupel system. In agreement with previous studies, these findings support the biological heterogeneity of Patnaik grade II MCTs, as intermediate differentiation does not reliably discriminate or predict tumor behavior (8, 39). Therefore, the combined assessment of both grading systems may improve histological classification (40).

Clinicopathological, histopathological, and tumor microenvironment-related variables were evaluated in all MCTs. Tumor necrosis due to hypoxia probably reflects an imbalance between the increased metabolic demand of neoplastic cells and insufficient oxygen supply and has been associated with higher histological grades (40, 41), in line with the results of the present study. Similarly, vascular invasion was associated with higher tumor grades in both classifications, as reported in other neoplasms, including canine anal sac apocrine gland adenocarcinoma (42), mammary carcinoma, and urothelial carcinoma (43). This finding is biologically plausible, since matrix metalloproteinases (MMPs) secreted by tumor cells promote extracellular matrix degradation, thereby facilitating local invasion and vascular dissemination (44).

The mitotic index is an indirect measure of cell proliferation based on the quantification of mitotic figures (45). In the present study, it was associated with higher tumor grade in both classification systems, consistent with previous reports (29, 45). According to the Patnaik system, tumors larger than 3 cm, incomplete surgical margins, and lymph node involvement were also associated with higher histological grade. Tumor size is unlikely to be a direct cause of malignancy; rather, larger tumors may reflect longer growth duration, greater tumor burden, clonal selection, and the accumulation of alterations that favor local invasion and metastatic dissemination. Accordingly, tumors larger than 3 cm have been associated with a higher frequency of lymph node involvement and greater aggressiveness (46), in agreement with our findings. Incomplete excision may reflect the infiltrative growth pattern of more aggressive tumors, characterized by poor delimitation and microscopic extension beyond the visible tumor margins (7, 8, 47). In line with previous literature, our findings also suggest that subcutaneous MCTs may show less aggressive biological behavior than cutaneous MCTs, although this result should be interpreted cautiously (48).

As part of innate immunity, eosinophils perform effector, tissue remodeling, cell-interaction, and immunomodulatory functions. In the tumor context, they may influence the microenvironment through the release of cytokines and granule proteins, as well as through interactions with tumor cells and other TME components, thereby affecting neoplastic progression and outcome (49–51).

The association between eosinophilia and malignancy was first reported in the 19th century by Rheinbach, who described marked blood eosinophilia in association with cancer. TATE was subsequently described in human neoplasms (52). However, the role of eosinophils in cancer and within the tumor microenvironment remains incompletely understood (51). The interpretation of TATE should consider the heterogeneity of the tumor microenvironment at several levels, including intertumoral, intratumoral, and organ-dependent variability (11). In early stages of tumor development, when eosinophilic infiltration is limited, eosinophils appear to exert antitumor functions by contributing to immune surveillance and promoting a Th1-type response. Conversely, in more advanced stages, increased eosinophilic infiltration may shift the Th1/Th2 balance toward a Th2-dominant profile, thereby favoring tumor progression (53). Moreover, in certain contexts, eosinophils may be recruited to tumor tissue through mechanisms independent of conventional chemokines (51).

The interaction between mast cells and eosinophils is well established and is particularly relevant in MCTs. These cell populations participate in a self-reinforcing biological loop, in which eosinophils promote mast cell activation and survival, whereas activated mast cells enhance eosinophil recruitment and activation, at least partly through mediators such as IL-5 and GM-CSF. This interaction may contribute to modulating tumor behavior and reinforcing specific tumor microenvironmental processes (19, 54, 55).

Despite the extensive information available on MCTs, further studies are still needed to better understand their biological complexity, as histological grading alone does not always adequately reflect tumor behavior (56). The integration of additional variables related to the tumor microenvironment may provide complementary information. Among these, TATE has been widely studied in human oncology, where it has been associated with both favorable (57) and unfavorable effects (58), depending on tumor type and biological context (59). The results of the present study support the hypothesis that the eosinophilic component of the tumor microenvironment may influence MCT behavior, as a higher eosinophil presence was associated with greater histological aggressiveness in the canine MCTs analyzed.

To the authors’ knowledge, this is the first study to specifically evaluate SII in canine MCTs. The interest of SII lies in its integration of platelets, neutrophils, and lymphocytes into a single variable (60). In the present study, SII was significantly associated with the Patnaik classification, with higher values observed in higher-grade MCTs. However, despite this statistical significance, the magnitude of the association appeared limited, as the OR was close to unity. Therefore, this finding should be interpreted cautiously and should not be considered evidence of a strong biological or clinically relevant association. Nevertheless, it is noteworthy that moderate-to-extensive intratumoral inflammation was significantly associated with Patnaik grade III tumors, suggesting that local inflammatory changes within the tumor microenvironment may be more closely related to histological aggressiveness than systemic inflammatory indices alone. Further studies including larger cohorts are needed to determine whether SII may be useful as a complementary marker in the biological characterization of canine MCTs.

The use of the EMR is supported by previous studies suggesting that relative values between immune populations within the tumor microenvironment may be more informative than isolated absolute counts (61–64). In this context, the EMR may represent a novel approach to exploring, in a more integrated manner, the interaction between the eosinophilic inflammatory component and the neoplastic mast cell population, as well as its potential contribution to the antitumor or protumor balance. Notably, our findings are consistent with those of Galietta (9), who reported that higher TATE scores were associated with less differentiated MCTs. This agreement is particularly relevant because tissue eosinophilia was expressed differently in the two studies: as absolute eosinophil counts in Galietta (9) and as the eosinophil-to-mast cell ratio in the present study.

The magnitude of the association between EMR and Kiupel grade should nevertheless be interpreted cautiously. The conventional logistic regression model produced a very large odds ratio with a wide confidence interval, suggesting limited precision and possible sparse-data-related inflation of the effect estimate. After applying Firth’s penalized logistic regression, the magnitude of the association was attenuated, although EMR remained strongly and statistically significantly associated with high-grade classification. Nevertheless, the very large effect estimates and wide confidence intervals suggest that the magnitude of the association should be interpreted with caution. These findings suggest that the exact magnitude of the effect is imprecisely estimated, whereas the direction and presence of the association appear robust across modeling approaches. For the Patnaik analyses, the proportional odds assumption was satisfied for all predictors in the primary model. In the alternative model, tumor type showed a non-proportional effect and was therefore modeled using a partial proportional odds approach. Importantly, EMR satisfied the proportional odds assumption in both models and remained significantly associated with increasing Patnaik grade.

Internal bootstrap validation provided further information on the stability of the exploratory EMR threshold. Although the median bootstrap-derived cutoff was identical to the original data-derived threshold of 0.38, the bootstrap distribution showed variability in the optimal cutoff (95% percentile interval, 0.29–0.63). Thus, the discriminatory performance of EMR appeared relatively stable, as reflected by the median bootstrap AUC of 0.796, whereas the exact threshold was less precisely determined. Accordingly, the cutoff of 0.38 should be considered exploratory and requires external validation before clinical implementation.

In this study, 98.3% of dogs had peripheral blood eosinophil counts within the reference interval, and this parameter was not associated with MCT grade. Although peripheral eosinophilia has been described in dogs with neoplasia, including MCTs (65), its occurrence appears to be variable. Isolated cases of marked hypereosinophilia have been reported, mainly in association with aggressive or metastatic clinical presentations (66). However, in a canine MCT study with a sample size similar to that of the present work, the incidence of peripheral eosinophilia was also low, below 10% (24). Importantly, the absence of peripheral eosinophilia does not exclude a local role for eosinophils within the tumor microenvironment, as TATE and peripheral blood eosinophil counts may reflect distinct biological compartments (67).

Although isolated absolute leukocyte counts may be insufficient to detect subtle alterations in the systemic inflammatory response, hematological ratios may provide complementary information by offering a more integrated view of systemic inflammation (60). Indices such as NLR, NER, and LMR may better reflect the interaction between the tumor and host response. Accordingly, increased NLR values have been associated with higher histological grades (24, 25). Although this ratio did not reach statistical significance in the present study, NLR tended to increase with tumor grade in both classification systems. A higher NLR may reflect an imbalance between systemic inflammation and antitumor immunity, with a relative predominance of neutrophils linked to protumor processes and a lower proportion of lymphocytes, suggesting a less effective cellular immune response (68).

To the authors’ knowledge, this is the first study to specifically evaluate the SII in canine MCTs. The interest of SII lies in its integration of platelets, neutrophils, and lymphocytes into a single variable (60). In the present study, SII was significantly associated with the Patnaik classification, with higher values observed in higher-grade MCTs. However, despite this statistical significance, the magnitude of the association appeared limited, as the OR was close to unity. Therefore, this finding should be interpreted cautiously and should not be considered evidence of a strong biological or clinically relevant association. Further studies including larger cohorts are needed to determine whether SII may be useful as a complementary marker in the biological characterization of canine MCTs.

Canine mast cell tumors are well-recognized for their pronounced intratumoral heterogeneity. Thus, a limitation of the present study is the absence of serial sectioning or systematic multi-region sampling of the tumors. Parameters such as necrosis, vascular invasion, mitotic index, and even eosinophil distribution may vary substantially across different regions of the same mass. Reliance on a limited set of sections (or consecutive high-power fields) may have led to an under- or overestimation of both the histological grade and the eosinophil-to-mast cell ratio (EMR) for a given tumor and limit the generalizability of these findings. Another potential limitation of the study is the lack of adjustment for within-dog clustering during the statistical analyses. Because approximately one-third of the dogs had multiple tumors, this may have influenced the estimated confidence intervals and the statistical significance of the results. Future multicenter studies incorporating complete serial sectioning or targeted sampling of macroscopic heterogeneous areas will contribute to further confirm the relationship between tumor-associated tissue eosinophilia and histological grade in canine mast cell tumors.

## 5 Conclusion

Given that the available oncological evidence on TATE may be conflicting and may differ according to tumor type, this study analyzed the numerical relationship between eosinophils and mast cells. Our results showed that a high EMR, defined as EMR > 0.38, was associated with high-grade MCTs. TABE was not associated with tumor grade, and most peripheral inflammatory markers showed no significant or clinically relevant relationship with MCT aggressiveness.

## Data Availability

The raw data supporting the conclusions of this article will be made available by the authors, without undue reservation.
